# Pre-bilaterian origin of the blastoporal axial organizer

**DOI:** 10.1038/ncomms11694

**Published:** 2016-05-27

**Authors:** Yulia Kraus, Andy Aman, Ulrich Technau, Grigory Genikhovich

**Affiliations:** 1 Department for Molecular Evolution and Development, Centre of Organismal Systems Biology, University of Vienna, Althanstraße 14, Vienna A-1090, Austria; 2 Department of Evolutionary Biology, Biological Faculty, Moscow State University, Leninskiye gory 1/12, Moscow 119234, Russia

## Abstract

The startling capacity of the amphibian Spemann organizer to induce naïve cells to form a Siamese twin embryo with a second set of body axes is one of the hallmarks of developmental biology. However, the axis-inducing potential of the blastopore-associated tissue is commonly regarded as a chordate feature. Here we show that the blastopore lip of a non-bilaterian metazoan, the anthozoan cnidarian *Nematostella vectensis*, possesses the same capacity and uses the same molecular mechanism for inducing extra axes as chordates: Wnt/β-catenin signaling. We also demonstrate that the establishment of the secondary, directive axis in *Nematostella* by BMP signaling is sensitive to an initial Wnt signal, but once established the directive axis becomes Wnt-independent. By combining molecular analysis with experimental embryology, we provide evidence that the emergence of the Wnt/β-catenin driven blastopore-associated axial organizer predated the cnidarian-bilaterian split over 600 million years ago.

## Abstract (web summary)

In vertebrate embryos, Wnt/β-catenin signaling induces an organizer area guiding the formation of body axes and inducing extra axes upon transplantation. Here, Kraus *et al*. show that Wnt ligands also induce an organizer in a sea anemone, indicating that the organizer dates back over 600 million years.

## Graphical abstract

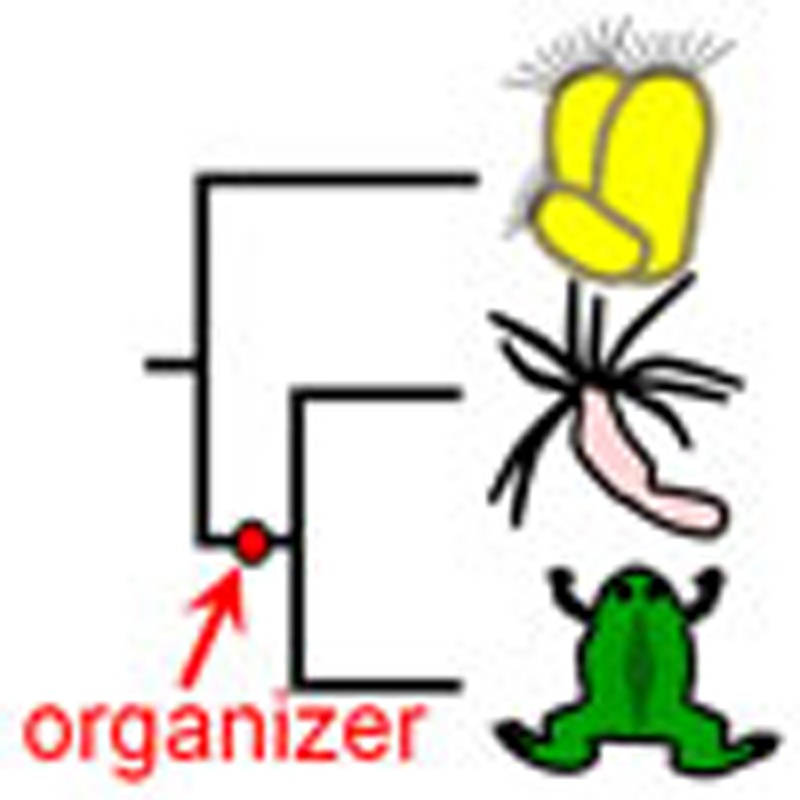

The ability of the amphibian dorsal blastopore lip to induce ectopic body axes upon transplantation has fascinated biologists for nearly a century^1^. Although axis-forming blastoporal organizers were long thought to be a chordate-specific feature, transplantation of a fragment of the mid-gastrula blastopore lip in the sea anemone *Nematostella vectensis*, a member of the early branching non-bilaterian phylum Cnidaria (which, in addition to sea anemones, includes corals, hydroids and jellyfish), also resulted in the formation of an ectopic body axis^2^, just like the Mangold-Spemann organizer in amphibians. Whether this similarity reflects the homologous or convergent origin of the cnidarian and vertebrate blastoporal axial organizers remained unclear, since the molecular nature of the signal conveying axial organizer properties to an embryonic tissue has not been determined outside deuterostomes.

In the frog *Xenopus*, the Spemann organizer expresses Wnt and BMP antagonists^3^, whose microinjection in single vegetal blastomeres can induce ectopic rostral or dorsal structures correspondingly^4^,^5^. However, complete ectopic body axes, like the ones seen during transplantation of the dorsal blastopore lip^1^ or the Nieuwkoop center cells^6^, nearly never form. In contrast, complete ectopic body axes can be induced by single blastomere injection of *Wnt-1, Xwnt-8*, *dishevelled*, *β-catenin* and dominant negative *GSK3β* mRNA^7^,^8^,^9^,^10^, showing that Wnt/β-catenin signaling is crucial for setting up the organizer. Pharmacological activation of Wnt/β-catenin signaling in *Nematostella* larvae affects the oral-aboral patterning, as detected by changes in the expression of *Wnt2*, *Wnt4*, *brachyury* (*Bra*) and several other genes^11^,^12^,^13^. Moreover, it also results in the formation of double-headed polyps^14^, making canonical Wnt signaling the prime candidate for the role of the early axial inducer in *Nematostella*, similar to the situation in frog. We set out to test whether Wnt signaling is conveying inductive capacity to the blastoporal organizer of *Nematostella*.

In this paper, using a combination of experimental embryology and molecular analyses, we show that two *Nematostella* Wnt ligands are capable of conveying axial organizing capacity to any region of the gastrula ectoderm, where they are co-expressed. Our data suggest an ancient origin of the Wnt/β-catenin dependent blastopore-associated axial organizer, predating the cnidarian-bilaterian split.

## Results

### Wnt1 and Wnt3 convey axial organizer capacity

**Figure 1:**
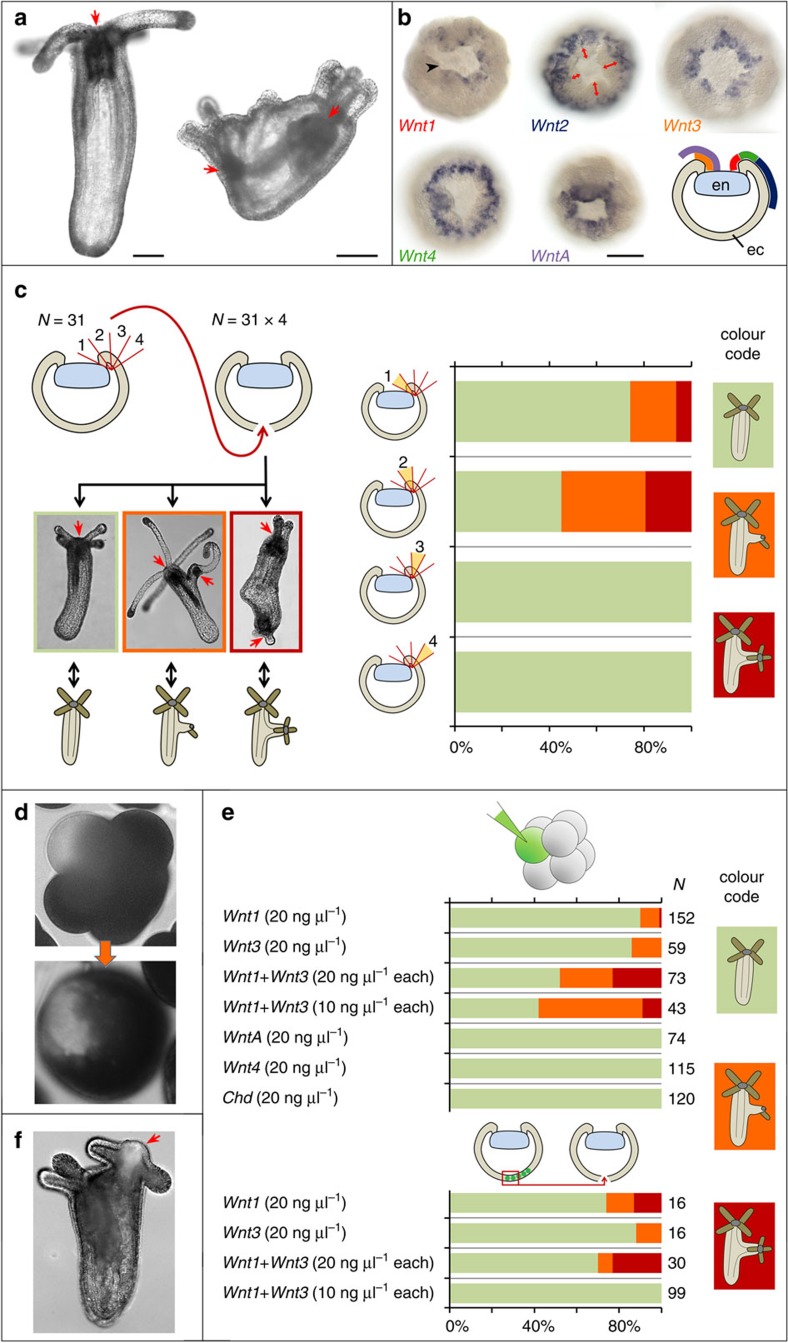
Ectopic expression of *Wnt1* and *Wnt3* induces ectopic body axes. (**a**) CRISPR-Cas9 knockout of *APC* results in the formation of ectopic tentacles and pharynges in F0. Left: wild type polyp; right: mosaic mutant polyp. Red arrows—pharynges. (**b**) Five ectodermal *Wnt* genes are expressed at mid-gastrula, however, *Wnt2* is expressed at a distance to the bend of the blastopore lip (red double-headed arrows). Gene names are colour-coded as on the scheme showing a lateral view on a mid-gastrula with ectodermal Wnt expression domains depicted as coloured lines. en—invaginating endoderm, ec—ectoderm. Black arrowhead points at the bend of the blastopore lip. (**c**) Results of transplantation of four sequential blastopore lip fragments from donor gastrulae (*N*=31) to four different recipient gastrulae (*N*=31 × 4). Possible developmental outcomes: no axis duplication (green bars on graph), incomplete axis duplication (an outgrowth with tentacles and, sometimes, pharynx but without mesenteries; orange bars on graph), complete axis duplication (two contractile axes with head structures; red bars on graph). Red arrows—pharynges. Fragment 1 and fragment 2, closest to the bend of the blastopore lip, are inductive. (**d**) Co-injection of a plasmid with *EF1α* promoter driving the expression of a gene of interest and Dextran-Alexa488 into single cells in 8–16 cell stage embryos results in formation of a coherent patch of fluorescent cells. (**e**) Some embryos injected into single blastomeres at 8–16 cell stage with *Wnt1* or *Wnt3* or with both these *Wnt*'s develop ectopic axes. *WntA*, *Wnt4* and *Chd* never induce a second axis. Fluorescent aboral ectoderm from *Wnt1* and *Wnt3* injected embryos also acquires inductive capacity. The colour code of the bars is the same as on (**c**). (**f**) If *EF1a::mOrange2* is co-injected with the *Wnt* plasmids, mOrange2 fluorescence is observed in the induced secondary head (red arrow). Scale bars: 100 μm.

We addressed the role of Wnt signaling in axis formation in *Nematostella* by performing CRISPR/Cas9-mediated knockout of the putative intracellular Wnt/β-catenin signaling inhibitor *APC*. Primary polyps mosaic for the *APC* mutation formed multiple ectopic tentacles and oral openings in F0 (Fig. 1a, Supplementary Fig. 1) suggesting that ectopic activation of Wnt/β-catenin signaling in discrete cell patches is sufficient for the induction of head structures. The *Nematostella* genome harbors 13 Wnt genes, which are expressed in staggered domains along the oral-aboral axis of the embryo and larva^15^,^16^. Since only the blastopore lip is capable of inducing a second axis upon transplantation^2^, our search for a candidate Wnt was restricted to *Wnt* genes, which are expressed in the blastopore lip of the *Nematostella* mid-gastrula. Five *Wnt* genes are expressed in concentric, partially overlapping ectodermal rings around the blastopore at mid-gastrula: *Wnt1*, *Wnt2*, *Wnt3*, *Wnt4* and *WntA* (Fig. 1b). To further narrow down the list of candidates, we subdivided the donor blastopore lips into four minute fragments along the oral-aboral axis and tested whether a certain part of the lip is particularly efficient in inducing secondary axes. We found that fragments immediately next to the bend of the lip were the most potent inducers (Fig. 1c). These fragments express *Wnt1*, *Wnt3, WntA* and, possibly, *Wnt4*, but not *Wnt2*, making the first four molecules the candidates for the role of head inducers. We then injected plasmids, where each of the four candidate Wnt genes was placed downstream of the *EF1α* promoter, into single blastomeres of the embryos at 8- or 16-cell stage. Dextran-Alexa488 fluorescence shows (Fig. 1d) that such injections result in the formation of a randomly located coherent patch of Wnt-expressing cells. While injections of 20 ng μl^−1^ solution of *Wnt4* and *WntA* plasmids were unable to induce the formation of the secondary axis, *Wnt1* and *Wnt3* plasmid injections resulted in the formation of incomplete secondary axes in 9–14%, respectively (Fig. 1e, Supplementary Fig. 2). An incomplete secondary axis represents a lateral outgrowth with tentacles and, sometimes, pharynx, but not connected with the mesentery system of the polyp. Interestingly, co-injection of *Wnt1* and *Wnt3* (both, at 20 and 10 ng μl^−1^ each) led to a much stronger induction, increasing the rate of ectopic axis formation to about 50%, including a large number of complete ectopic axes with tentacles, pharynx and contractile mesenteries connected with the mesenterial system of the host polyp (Fig. 1e, Supplementary Fig. 2). Moreover, transplantation of fluorescent fragments of the aboral tissue ectopically expressing *Wnt1* and/or *Wnt3* was able to induce formation of the secondary axes (in 30% of the cases), while this part of the embryo is absolutely devoid of inductive capacity in the uninjected control embryos (Fig. 1e). Similar to the frog embryo, the blastopore lip of the *Nematostella* early and mid-gastrula also expresses BMP antagonist *chordin* (*Chd*)^17^. However, ectopic overexpression of *Chd* by single blastomere injection does not induce ectopic axes (*n*=0/120) (Fig. 1e). We conclude that Wnt/β-catenin signaling acts as head inducer in *Nematostella*, and Wnt1 and Wnt3 appear to act synergistically in conveying the inductive capacity to the blastoporal organizer tissue. Unfortunately, fluorescent dextran used as injection tracer is not detectable in the primary polyps. In order to test whether the progeny of the *EF1α::Wnt1/EF1α::Wnt3* injected blastomere becomes part of the induced ectopic axis, we added the *EF1α::mOrange2* plasmid to the injection mix and showed that the mOrange2-expressing tissue contributes to the pharynx and the hypostome of the induced secondary head (Fig. 1f).

### β-catenin signaling defines the location of the organizer

**Figure 2:**
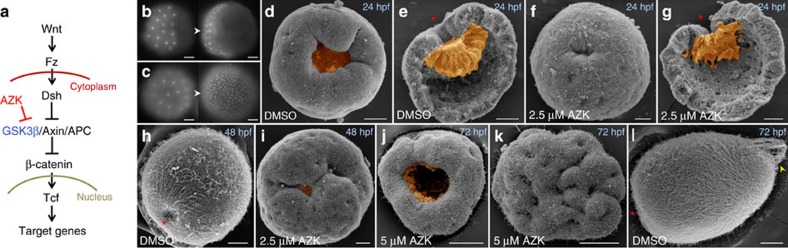
Early AZK treatment results in formation of severely oralized embryos. (**a**) AZK upregulates Wnt/β-catenin signaling by inhibiting GSK3β. (**b**) Nuclear translocation of β-catenin-venus fusion protein on one side in 6 and 9 h post fertilization (hpf) embryos injected with *β-catenin-venus* mRNA. (**c**) Nuclear β-catenin-venus in all cells at 6 and 9 hpf of AZK treated blastulae. (**d**,**e**) SEM of control 24 hpf mid-gastrulae; oral (**d**) and lateral (**e**) views. Note large blastopore and pre-endodermal plate. (**f**,**g**) Small blastopore and pre-endodermal plate on SEM of mid-gastrulae after early treatment with 2.5 μM AZK. Gastrulae on (**e**) and (**g**) were split into halves to make inner structures visible. (**h**,**i**) Control 48 hpf planula (**h** lateral view) and a planula subjected to the early 2.5 μM AZK treatment (**i**, oral view). In treated planula, blastopore starts to re-open and small pits appear throughout the surface. (**j**–**l**) oral (**j**) and aboral (**k**) views of the 72 hpf planula after early 5 μM AZK treatment and of the control 72 hpf planula (**l**). The oral surface of the treated embryo carries a re-opened blastopore (**j**), the aboral surface shows multiple folds and holes. The embryo fails to elongate and form aboral structures (e.g., apical tuft - yellow arrowhead on (**l**)). Scale bars: 30 μm. Endoderm highlighted orange. Red asterisks on lateral views denote blastopores.

**Figure 3:**
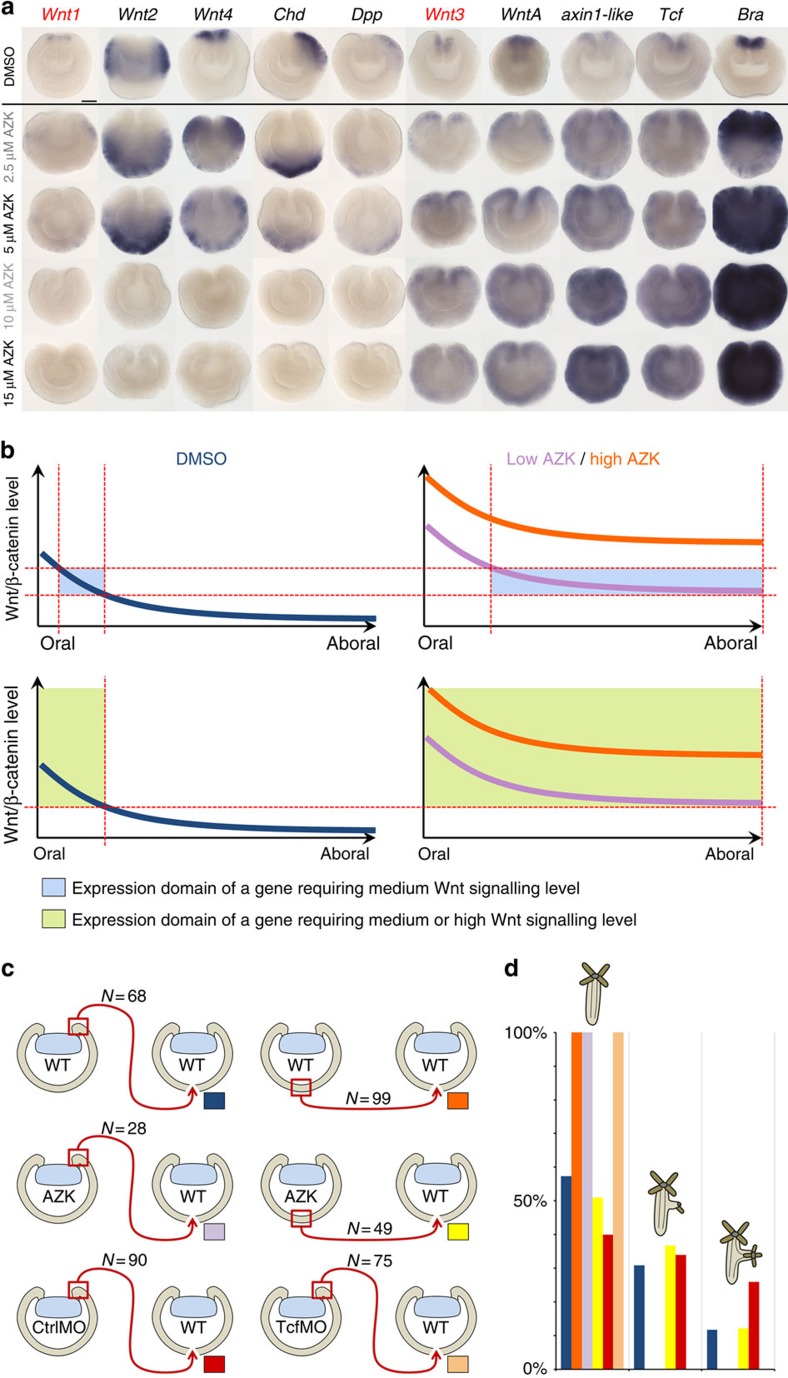
Organizer capacity shifts to follow changes in *Wnt1* and *Wnt3* expression. (**a**) Expression of *Wnt1*, *Wnt2*, *Wnt4*, *Chd* and *Dpp* expands aborally while clearing from the oral domain and disappears with the increasing AZK concentrations; expression of *Wnt3*, *axin1-like*, *Tcf* and *Bra* expands globally and reaches saturation. Blastopores point up; scale bar: 50 μm. (**b**) Model of expression of a gene activated at medium Wnt/β-catenin signaling levels (upper row; blue highlight represents the range of Wnt/β-catenin signaling levels when the gene can be expressed) versus a gene activated at medium and high Wnt/β-catenin signaling levels (lower row; green highlight represents the range of Wnt/β-catenin signaling levels when the gene can be expressed) in the AZK treatments. Blue curve: wild type level of Wnt/β-catenin signaling along the oral-aboral axis. Lilac curve: Wnt/β-catenin signaling level in a low concentration of AZK. Orange curve: Wnt/β-catenin signaling level in a high concentration of AZK. Red dashed lines mark the Wnt/β-catenin signaling thresholds and the corresponding positions on the oral-aboral axis where these thresholds are reached. (**c**,**d**) Scheme (**c**) and developmental outcomes (**d**) of transplantation of wild type (wt) and 2.5  μM AZK treated (AZK) blastopore lips and aboral ectoderm fragments as well as blastopore lips of the CtrlMO and TcfMO injected gastrulae into wild type recipient gastrulae. Coloured rectangles on (**c**) correspond to the colours of the bars on (**d**). Only untreated or CtrlMO blastopore lips and AZK treated aboral ectoderm fragments are inductive.

To get insight into the molecular response of the embryonic tissue and, particularly, the organizer to the elevated Wnt/β-catenin signaling in *Nematostella*, we modulated the Wnt/β-catenin signaling (Fig. 2a–c) by inhibiting GSK3β kinase with 1-Azakenpaullone (AZK) at different times during embryonic development. The treatment with 2.5 μM AZK from early cleavage to mid-gastrula (hereafter referred to as early treatment) resulted in the formation of gastrulae with smaller blastopores and pre-endodermal plates (Fig. 2d–g, Supplementary Fig. 3); however, the process of gastrulation was not perturbed. By day 2 (a stage corresponding to the early planula in controls), these AZK treated embryos flattened and their blastopore openings expanded (Fig. 2h,i). If higher concentrations of AZK were used (5 μM or more), additional openings appeared on their aboral sides (Fig. 2j–l, Supplementary Fig. 3). These severely oralized embryos did not develop further and died after several days. We then assayed the expression of the ectodermal *Wnt* genes and the putative Wnt signaling targets *axin1-like, Tcf* and *Bra* in the gastrulae subjected to early treatment with different concentrations of AZK. While 1 μM AZK treatments did not cause noticeable change in the expression of all the assayed genes, the treatments with 2.5, 5, 10 and 15 μM AZK showed a clear difference in the reaction of these genes to AZK treatment. *Wnt3*, *WntA*, *axin1-like*, *Tcf* and *Bra* were upregulated throughout the embryo (Fig. 3a, Supplementary Fig. 4, Supplementary Table 1), suggesting that these genes are activated by medium as well as high level of Wnt/β-catenin signaling (Fig. 3b). In contrast, in increasing AZK concentrations, *Wnt1*, *Wnt*2 and *Wnt4* started to be upregulated in more and more aboral positions, while vacating the oral domain and finally disappearing from the embryo (Fig. 3a, Supplementary Fig. 4, Supplementary Table 1). This finding suggests that *Wnt1*, *Wnt*2 and *Wnt4* are activated by medium levels but repressed, either directly or indirectly, by high levels of Wnt/β-catenin signaling (Fig. 3b). To control for the possibility that the observed changes in the expression were due to the described off-target effect of high concentrations of AZK on cyclin dependent kinase 1 (CDK1) rather than on GSK3β^18^, we treated the embryos with the CDK1/CDK2/CDK5 inhibitor Aminopurvalanol A (AMPV) in the same time window as the early AZK treatment, i.e., from early cleavage to mid-gastrula. As expected, higher concentrations of AMPV arrested the development, either immediately after addition of the drug (5 or 10 μM AMPV) or several cell cycles later (2 μM AMPV), and resulted in the death of the embryos by the time gastrulation had to start. We then used a sub-lethal concentration of AMPV (1 μM), in which the embryos were able to gastrulate, and performed α-phosphohistone H3 antibody staining with 1 μM AMPV treated embryos, 15 μM AZK treated embryos and DMSO control embryos at the gastrula stage. Metaphase plates were observed in all three treatments (Supplementary Fig. 5a), however no shifts in the expression domains of *Wnt2*, *Wnt4* and *Bra* could be detected in the AMPV treated embryos (Supplementary Fig. 5b), suggesting that the observed effects of the AZK treatment on gene expression (Fig. 3a) were due to the inhibition of GSK3β.

To test whether the change of expression domains of *Wnt1* and *Wnt3* in AZK treated embryos correlated with the changes in the inductive capacity of the gastrula tissue, we performed transplantations using donor embryos subjected to the early treatment with 2.5 μM AZK. Strikingly, and concomitant with the loss of *Wnt1* expression in the blastopore lip in the AZK treated embryos, transplanted blastopore lips of the AZK treated embryos were completely unable to induce secondary axes in the recipients (Fig. 3c,d). However, the normally non-inductive aboral tissue, which in the AZK-treated embryos represents the tissue co-expressing *Wnt1* and *Wnt3* (Fig. 3a), could induce ectopic axis formation in the recipients just like the wild type blastopore lips (Fig. 3c,d), suggesting a shift of the organizer capacity from the blastopore lip to an aboral location. Further supporting the role of Wnt/β-catenin signaling in organizer formation, morpholino knockdown of Tcf, the conserved transcriptional co-factor of β-catenin, abolished the inductive capacity in the morphant blastopore lip (Fig. 3c,d).

### Establishment of the inductive territory

**Figure 4:**
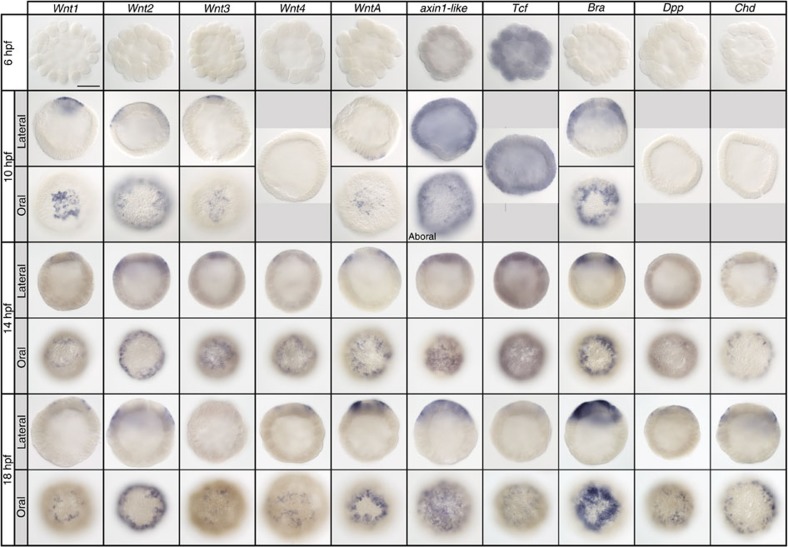
Establishment of the oral inductive territory in *Nematostella*. At 10 hpf *Wnt1*, *Wnt2*, *Wnt3* and *WntA* are the first *Wnt* genes to be expressed. *Wnt2* expression starts as a ring excluding the presumptive pre-endodermal plate. *Wnt1*, *Wnt3* and WntA expression starts as a patch in the presumptive pre-endodermal plate and by 14–18 hpf begins to form a ring around it. *Brachyury* expression is first detectable as a ring at 10 hpf. *Wnt4*, *Dpp* and *Chd* start to be expressed in a ring around the pre-endodermal plate by 14 hpf. On lateral views, animal/oral ends of the embryos point up. Scale bar: 100 μm.

Our data clearly show that axial organizer formation in *Nematostella* is a Wnt/β-catenin signaling dependent process. It has been previously demonstrated that nuclear β-catenin starts to accumulate at the future oral side of the *Nematostella* embryo already during early cleavage^19^ and that Dishevelled protein is localized at the animal pole of the unfertilized egg^20^. To get further insight into the establishment of the future inductive territory during development, we analyzed the expression of *Wnt1*, *Wnt2*, *Wnt3*, *Wnt4*, *WntA*, *axin1-like*, *Tcf* and *Bra* from early blastula to pre-gastrula in the wild type embryos (Fig. 4). *Tcf*, is strongly expressed in the 6 h post fertilization (hpf) blastula (although we did not see an accumulation at the future oral pole as previously suggested^16^), and *axin1-like*, is weakly expressed at 6 hpf. In contrast, the ectodermal *Wnt* genes (except *Wnt4*) become detectable in 10 hpf blastulae, together with *Bra* (Fig. 4). Interestingly, *Wnt1*, *Wnt3* and *WntA* are first expressed in a patch of cells and only later refine into a ring, while *Wnt2* and *Bra* are detected as a ring from the start. The early expression patterns of *Wnt1*, *Wnt3* and *WntA* suggest that they are either involved in the activation of the genetic programme defining the pre-endodermal plate or co-regulated by the same early β-catenin input that activates the endoderm determination programme throughout Eumetazoa^19^,^20^,^21^,^22^,^23^,^24^,^25^. Once the endodermal territory is determined, *Wnt1*, *Wnt3* and *WntA* expression becomes displaced out of the prospective endodermal cells, likely by a yet unknown negative feedback mechanism, and becomes confined to the ectodermal tissue surrounding the endoderm. These ectodermal cells will become the axial organizer.

### Wnt/β-catenin signaling and the directive axis

**Figure 5:**
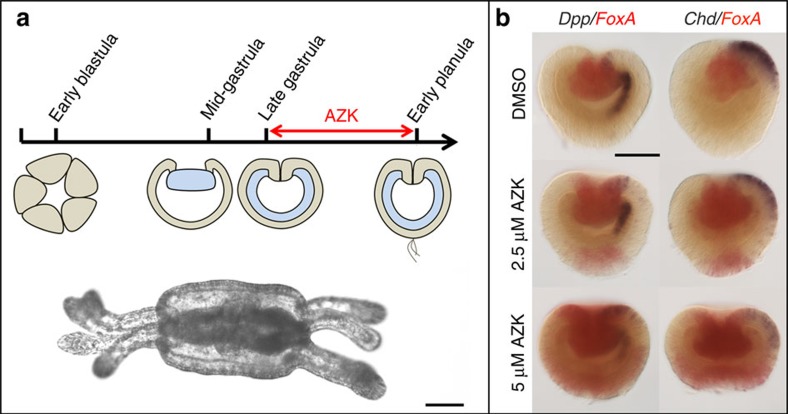
Late AZK treatment affects the oral-aboral but not the directive axis. (**a**) Scheme of late AZK treatment and a resulting double-headed primary polyp. (**b**) An ectopic domain positive for the oral marker *FoxA* appears on the aboral end of the planula subjected to late AZK treatment, however, unilateral expression of *Dpp* and *Chd* in treated embryos is retained. Blastopores point up. Scale bars: 100 μm.

In the vertebrate organizer, the initial β-catenin signal activates the expression of BMP antagonists such as *chordin*, thus suppressing ventral fates^3^. Anthozoan cnidarians, unlike their radially symmetric hydroid and jellyfish relatives, possess not only the oral-aboral body axis but also a second, directive axis, orthogonal to the oral-aboral axis. The key players for the establishment of the directive axis are the *bmp4* homologue *Dpp* and the *chordin* homologue *Chd*^26^. Their initially radial expression starts at 14 hpf (Fig. 4) and shifts to the same side of the directive axis upon BMP signaling-dependent symmetry break in late gastrula^17^,^26^. It has been previously shown that *Dpp* and *Chd* is negatively affected by suppression of Wnt/β-catenin signaling by iCRT14 (ref. 11), and microarray analysis showed that both genes react to 10 μM AZK^13^. Interestingly, the expression of *Dpp* and *Chd* reacted to the early AZK treatment in a similar way as the expression of *Wnt1*, *Wnt2* and *Wnt4*, displaying an aboral expansion at lower AZK concentrations and disappearing at higher AZK concentrations, indicative of the lack of directive axis (Fig. 3a, Supplementary Fig. 5). The disappearance of *Chd* expression was previously described^11^ for a different GSK3β inhibitor, alsterpaullone (ALP), although only one concentration of ALP was used. Surprisingly, we did not observe the reported upregulation^11^,^13^ and ubiquitous expansion^11^ of *Dpp* expression in early AZK treatment. The effect of the early AZK treatment is in stark contrast to the effects of the late AZK treatment (24 h incubation in 2.5 or 5 μM AZK starting from late gastrula), which we performed after the time of blastoporal organizer formation and directive axis establishment. Late AZK treatment did not prevent larva development and primary polyp formation, but resulted in the oral transformation of the aboral end of the embryo. Notably, such embryos never developed ectopic axes laterally, but rather re-specified the aboral half of their oral-aboral axis to acquire oral fates (Fig. 5a). Strikingly, late AZK treatment did not affect the asymmetric expression of *Dpp* and *Chd* (Fig. 5b, Supplementary Fig. 4, Supplementary Table 2), while the expression of the oral marker *FoxA*^27^ clearly demonstrated the transformation of the aboral domain into an oral domain (Fig. 5b). These observations suggest that the initial, radially symmetric, circumblastoporal expression of *Dpp* and *Chd* might rely on the correct intensity of β-catenin signaling, however, *Dpp* and *Chd* expression becomes self-regulating and does not require Wnt/β-catenin input, once the directive axis is established.

## Discussion

In this paper, we showed that the key molecules conveying the inductive capacity to the *Nematostella* organizer are *Wnt1* and *Wnt3*. Their co-expression in any region of the ectoderm of the gastrula following the single blastomere injection appears to be sufficient for the tissue to acquire axial organizer properties. If expression domains of Wnt/β-catenin signaling dependent genes in the mid-gastrula are modulated by AZK treatment, the inductive capacity is lost in the blastopore lip and becomes confined to the aboral ectoderm, correlated with the shifts in the *Wnt1* and *Wnt3* expression. Our analysis of the onset of *Wnt1* and *Wnt3* expression in the wild type *Nematostella* embryos showed that their early expression is first observed not in a ring, but in a patch of cells in the 10 hpf blastula, and only later it starts to refine into a ring surrounding the presumptive pre-endodermal plate. It seems reasonable to predict that transplantation of a patch of *Wnt1* and *Wnt3* expressing cells from a 10 hpf blastula (Fig. 4) into an aboral position of a recipient embryo would also lead to axis induction. Currently, due to the lack of morphological landmarks in the blastula, such experiment is not possible, however, once transgenic lines expressing a fluorescent reporter under control of *Wnt1* or *Wnt3* regulatory elements are generated, it will be feasible to test whether these early presumptive endodermal cells expressing *Wnt1* and *Wnt3* already have the inductive capacity, reminiscent of the Nieuwkoop center cells^6^, which are also located in the endodermal territory in the frog embryo.

**Figure 6:**
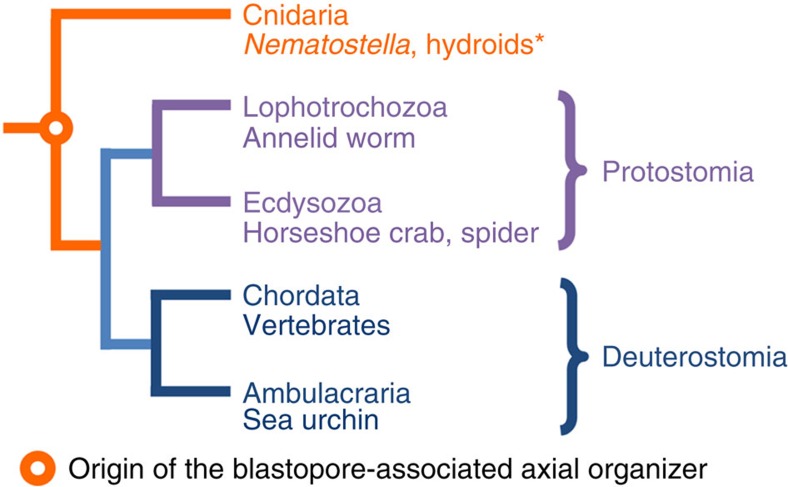
The origin of the blastopore-associated axial organizer. In hydroids (marked by *), organizing capacity is detected in the oralmost tissue: the swimming posterior tips of the planula larvae and the hypostomes of polyps.

In summary, Wnt/β-catenin signaling dependent blastopore lip organizers are capable of inducing twin new body axes in the animals evolutionary as far apart as cnidarian *Nematostella* and vertebrates. Axial organizer capacity has been reported for other cnidarians as well. The swimming posterior pole of hydrozoan planulae, which corresponds to the blastoporal end of the sea anemone planula, is capable of inducing ectopic axes^28^,^29^. Also, the hypostome (oral cone) tissue of the solitary hydroid polyp *Hydra*, as well as of its colonial relative *Clava*, has axial organizer capacity^28^,^30^, and this adult organizer capacity in *Hydra* is Wnt/β-catenin dependent^31^,^32^. Maybe due to the difficulty of performing transplantation experiments or because inductive capacity was not expected in embryos, whose body axes are established maternally, or whose development is invariant, little is known about the axial organizing capacity of the blastopore-associated tissue in non-chordate Bilateria. However, there is evidence suggesting that blastopore-associated axial organizers also exist in the non-chordate deuterostomes as well as in the ecdysozoan and lophotrochozoan protostomes. Grafting of the micromeres from the 16-cell sea urchin embryo from the vegetal pole into the animal position induces the formation of the twin gut and a complete second set of larval axial structures^33^. The sea urchin micromere lineage is characterized by strong nuclear β-catenin from the fifth cleavage cycle on^24^. Grafting of the center cells from the vicinity of the blastopore in a horseshoe crab, which is an early-branching chelicerate arthropod, results in the formation of larvae with complete duplicated axes^34^. The same effect is seen in cumulus transplantations in a spider^35^. Moreover, even in the oligochaete annelid *Tubifex*—displaying a highly invariant, cell lineage based development—grafting of the 2d^11^ and 4d blastomeres into an ectopic position also results in the formation of ectopic axes^36^, although concerns have been raised whether the grafted cells actually induce the host cells to contribute to the formation of the ectopic axis or whether they form the second axis autonomously^37^. A more recent blastomere ablation study on a different annelid, the polychaete *Capitella*, showed that the 2d somatoblast emits a signal required for trunk formation and the D-V axis organization in the head^37^, although the authors did not perform transplantations of the 2d cell, and the molecular nature of the signal remains unknown^37^. In general, the D-quadrant and, particularly, the 4d cell and its descendants in Spiralia are the site of nuclear β-catenin stabilization^21^,^22^. In the invaginating gastrulae of the species with homoquadrant cleavage these cells are located at the dorsal margin of the blastopore^38^. However, the possible role of β-catenin in the formation of axial organizers in protostome Bilateria requires further thorough analysis. Taken together, our study strongly suggests an ancient origin of the blastopore-associated axial organizer (Fig. 6) based on Wnt/β-catenin signaling, which was maintained in the cnidarian, deuterostome and, possibly, in several protostome lineages.

## Methods

### Animal culture and plasmid microinjection

Animal husbandry was performed as described^39^. Briefly, adult polyps were kept in *Nematostella* medium (16‰ artificial sea water) at 18 °C in the dark. Induction of spawning was performed by placing the animals into 25 °C illuminated incubator for 10 h. Developing embryos were kept at 21 °C. Plasmids carrying different untagged *Nematostella Wnt's* and *mOrange2* driven by *EF1α* promoter were injected^40^ into individual blastomeres at 8–16 cell stage. Fluorescent Dextran-Alexa488 (Life Technologies) was co-injected as tracer.

### CRISPR-Cas9 mediated mutagenesis

Single guide RNAs for CRISPR-Cas9 mediated mutagenesis of *Nematostella APC* (Genbank KT381584) were synthesized^41^ using the following oligos: 5′TAGGCACAGCTATGAGGGCCAC, 5′AAACGTGGCCCTCATAGCTGTG. nls-Cas9 protein was obtained from PNA bio (Thousand Oaks, CA, USA). 500 ng/μl single guide RNA and 1.5 μg/μl nls-Cas9 were injected into single cell embryos^42^. Control embryos were injected with 500 ng/μl single guide RNA alone. DNA was prepared from individual polyps and a 248 bp long fragment containing the recognition sequence of the guide RNA was amplified using the following oligos: 5′AGAATCCTGCAGAAGATGAACA, 5′ CCTGGCATACAAAGGTGACA. For the controls, 10 animals were pooled prior to DNA preparation. PCR products harboring the mutation site were directly sequenced by Microsynth (Balgach, Switzerland), and sequencing chromatograms were analyzed to assess the presence of the discrepancies in comparison to the wild type sequence.

### Inhibitor treatments and morpholino experiments

1-Azakenpaullone (Sigma) and Aminopurvalanol A (abcam) treatments were performed in the time windows and with concentrations specified in the main text. Tcf knockdown was performed by injecting a previously published antisense translation blocking morpholino^11^,^13^ (TcfMO; 5′ CTGAGGCATACTACCGTCTCATGTG) into fertilized eggs at 500 μM concentration. A morpholino recognizing the splice acceptor in front of the exon 9 of *Nvmef2*, which does not produce any noticeable developmental phenotype^43^, was used as control (CtrlMO; 5′ GATGTGCCTAGGGTACAACAACAAT) at the same concentration. Transplantation of different fragments of the gastrulae was performed as previously described^2^. Briefly, the fragments of the donor gastrula tissue were excised with a feather microscalpel (Science Services, blade angle 30° or 45°) and implanted into small windows cut out in the aboral pole of the recipient gastrulae under a dissecting scope (Nikon SMZ745). For transplantation of the fluorescently labeled tissue, a fluorescent dissecting scope (Nikon SMZ18) was used. During and after transplantation, the embryos were kept in *Nematostella* medium.

### *In situ* hybridization and morphological analysis

In situ hybridization was performed as described^44^. Briefly, embryos from cleavage stage till gastrula were fixed for 1 min in 2% glutaraldehyde/3.7% formaldehyde in *Nematostella* medium (NM) and then for an additional hour in 3.7% formaldehyde/NM. Older embryos and larvae were fixed for 1 min in 0.2% glutaraldehyde/3.7% formaldehyde in *Nematostella* medium and then for an additional hour in 3.7% formaldehyde/NM. After 5 PTw (1x PBS with 0.1% Tween 20) washes, the embryos were transferred into methanol, then rehydrated with PTw and treated for 3 min with 80 μg/ml proteinase K (Ambion). After stopping the digest with two 4 mg/ml Glycin/PTw washes, the embryos were washed four times in 1% triethanolamine/PTw for 5 min. The last two triethanolamine washes contained 3 μl/ml and 6 μl/ml of acetic anhydride. After two additional PTw washes, the samples were post-fixed for 1 h in 3.7% formaldehyde/PTw, washed 5x with PTw, prehybridized at 60 °C for 2 h in the Hybe buffer (50% formamide, 5 × SSC pH4.5, 1% SDS, 0.1% Tween 20, 50 μg/ml heparin, 5 mg/ml *Torula* yeast RNA) and then hybridized at 60 °C for 36–60 h in Hybe containing 0.5 ng/μl of the Digoxigenin or FITC-labeled antisense RNA probe against *Wnt1* (nucleotides 44–1135 of Genbank AY530300), *Wnt2* (full length Genbank AY725201), *Wnt3* (nucleotides 11–1051 of Genbank DQ492689), *Wnt4* (nucleotides 53–1114 of Genbank AY687348), *WntA* (nucleotides 35–1129 of Genbank AY534532), *Bra* (nucleotides 52–1222 of Genbank AF540387), *axin1-like* (nucleotides 1–1123 of Genbank JQ959548), *Tcf* (nucleotides 1–1191 of Genbank DQ497247), *Dpp* (nucleotides 134–1267 of Genbank AY363391), *Chd* (nucleotides 1768–2584 of Genbank DQ286294) or *FoxA* (nucleotides 233–1093 of Genbank AY457634). After gradually transferring the samples from Hybe into 2 × SSCT (2 × SSC with 0.1% Tween 20) in three 30 min steps at 60°C, the embryos were washed once for 30 min with 2 × SSCT and three times for 30 min with 0.05 × SSCT at 60 °C. Then the samples were briefly washed with PTw and blocked in 1% Blocking reagent (Roche) in MAB for 1 h at room temperature. Then the samples were incubated overnight at 4 °C with alkaline phosphatase conjugated Anti-Dig or Anti-FITC Fab fragments (Roche) diluted 1:2000 in the 1% Blocking reagent/MAB, washed 10 times for 15 min in PTw and twice for 5 min in the buffer for alkaline phosphatase and stained with NBT/BCIP (Roche) or Fast Red (Roche). Double in situ staining was performed as described^45^. Briefly, after staining overnight at 4 °C with anti-FITC Fab fragments conjugated with alkaline phosphatase, washing 10 times for 15 min in PTw and performing the first substrate reaction with Fast Red according to the protocol of the manufacturer, the samples were rinsed twice in PTw, and alkaline phosphatase was inactivated by a 10 min wash in 100 mM glycine-HCl (pH 2.2) at room temperature. Then, the samples were rinsed twice in PTw, re-blocked in 1% Blocking reagent/MAB, stained with the anti-Dig Fab fragments conjugated with alkaline phosphatase overnight at 4 °C, washed 10 times for 15 min in PTw and stained with NBT/BCIP. After staining, the samples were rinsed with water, washed with absolute ethanol (this step has to be done carefully if ethanol-sensitive Fast Red staining was performed), then rinsed with PTw and embedded in 86% glycerol. In situ patterns were imaged and quantified under a Nikon 80i compound microscope.

Semithin sectioning and SEM was performed as specified^46^. Briefly, the embryos were fixed in 2.5% glutaraldehyde/0.1 M cacodylate buffer (pH 7.2) and stored in 1.25% glutaraldehyde/0.1 M cacodylate buffer. Then they were washed with 0.1 cacodylate buffer, post-fixed for 1 h in 1% OsO_4_/0.1 M cacodylate buffer, washed, dehydrated in ethanol and acetone and either dried by critical point technique and sputter coated for SEM or embedded into a standard mixture of Araldite and Epon (Electron Microscopy Sciences) for semithin sectioning. 1 μm semithin sections were stained with 1% toluidine blue and imaged under the Nikon 80i compound microscope. SEM was performed with the JEOL JSM-6380LA or CamScan S2 microscopes.

### Antibody staining

Rabbit anti-phospho-Histone H3 (Ser10) antibody (Merck Millipore) was used in 1:400 dilution using the antibody staining protocol from^47^. Briefly, the embryos were fixed in 4% paraformaldehyde/PBS-TT (1xPBS, 0.2% Triton X100, 0.2% Tween 20), washed five times in PBS-TT, blocked in 20% heat inactivated sheep serum (Sigma)/1% BSA in PBS-TT, and incubated with the primary antibody overnight at 4 °C. After eight 15 min PBS-TT washes, the embryos were blocked again and incubated with the goat anti-rabbit Alexa568 (Life Technologies) at 1:1000 dilution overnight. DAPI was added at the final concentration of 300 nM together with the secondary antibody to counterstain chromatin. The samples were then washed again eight times 15 min with PBS-TT, embedded in Vectashield (Vectorlabs) and imaged under a Leica SP5X CLSM.

### Data availability

The data supporting the findings of this study are available within the article and its Supplementary Information. Any further relevant data concerning the techniques used in the paper are available on request.

## Additional information

**How to cite this article:** Kraus, Y. *et al*. Pre-bilaterian origin of the blastoporal axial organizer. *Nat. Commun.* 7:11694 doi: 10.1038/ncomms11694 (2016).

## Supplementary Material

Supplementary Information Supplementary Figures 1-5 and Supplementary Tables 1-2
