# Supplementary material for: Pre-bilaterian origin of the blastoporal axial organizer
Source: Nat Commun. 2016 May 27;7:11694. doi: 10.1038/ncomms11694 (PMC4895019; doi:10.1038/ncomms11694)
Supplement: Supplementary Information — Supplementary Figures 1-5 and Supplementary Tables 1-2 [file ncomms11694-s1.pdf]

Supplementary Figures

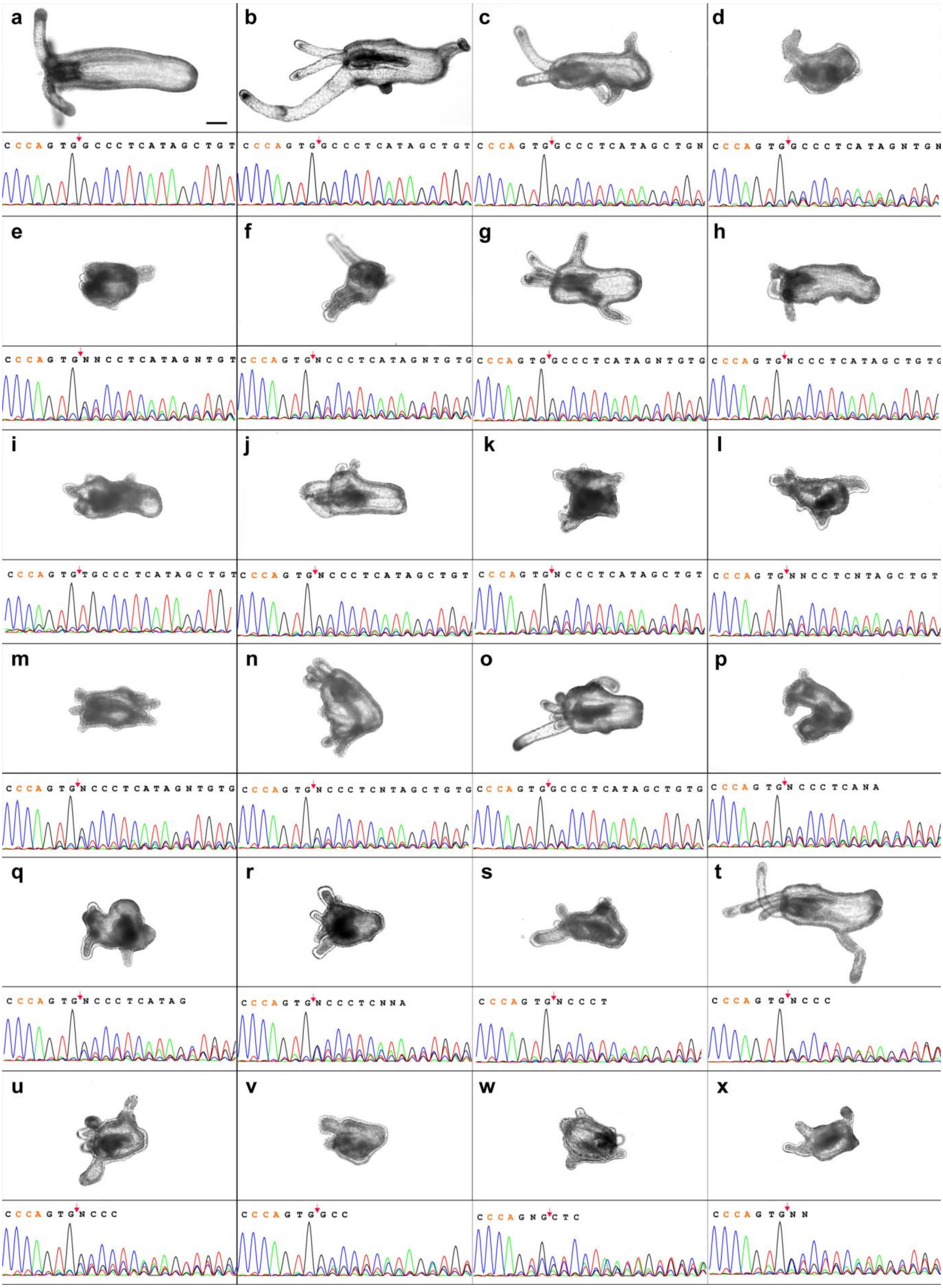

Supplementary Figure 1.

CRISPR-Cas9 mutagenesis of *APC* leads to formation of ectopic head structures in mosaic mutants. **(a)** Control primary polyp and the sequencing chromatogram of the locus recognized by the *APC* guide RNA from a pool of 10 control primary polyps injected with *APC* guide RNA alone. Note the absence of sequence variability in the region of interest. **(b-x)** Mutant primary polyps with ectopic head structures (tentacles, pharynges) and corresponding sequencing chromatograms of the *APC* guide RNA target locus. Note the positions with multiple peaks indicating the presence of mutations. PAM sequence is highlighted orange; red arrow points at the expected Cas9 cleavage site. Scale bar: 100 µm.

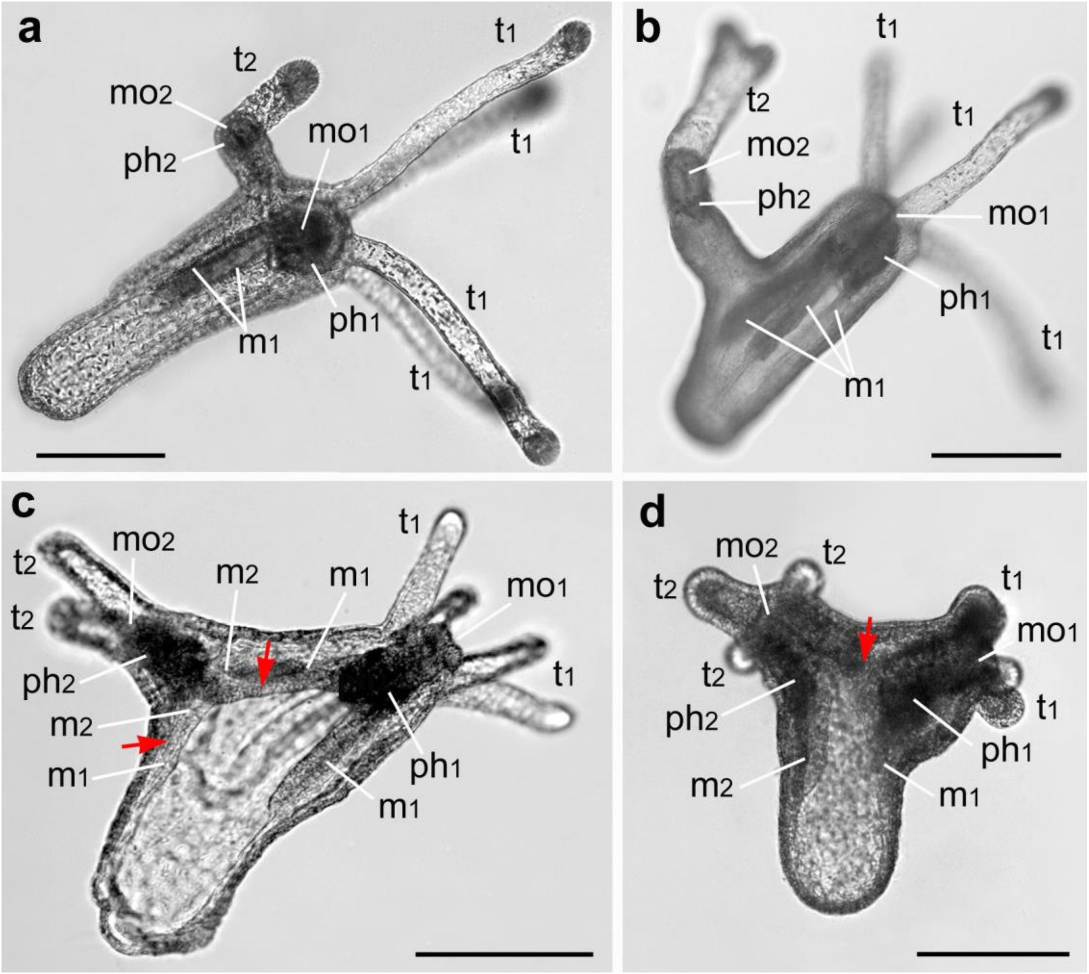

Supplementary Figure 2.

Blastopore lip transplantation and *Wnt1*/*Wnt3* plasmid injection yield similar outcomes.

(a-b) Examples of incomplete secondary axes with single tentacles, mouths, pharynges and no mesenteries in 7 days post-fertilization primary polyps after transplantation of a fragment of the blastopore lip at the mid-gastrula stage **(a)**, and after injection of a mixture of *EF1α::Wnt1* and *EF1α::Wnt3* into a single blastomere at the 8-cell stage **(b)**. **(c-d)** Examples of complete secondary axes with tentacles, mouths, pharynges and contractile mesentery systems in 7 days post-fertilization primary polyps after transplantation of a fragment of the blastopore lip at the mid-gastrula stage **(c)**, and after injection of a mixture of *EF1α::Wnt1* and *EF1α::Wnt3* into a single blastomere at 8-cell stage **(d)**. Annotated structures: tentacles on the main (t1) and ectopic (t2) body axes; pharynges on the main (ph1) and ectopic (ph2) body axes; mouth openings on the main (mo1) and ectopic (mo2) body axes; contractile

mesenteries on the main (m1) and ectopic (m2) body axes. Red arrows point at mesenteries belonging to both the main and the ectopic axes. Scale bars: 100 µm.

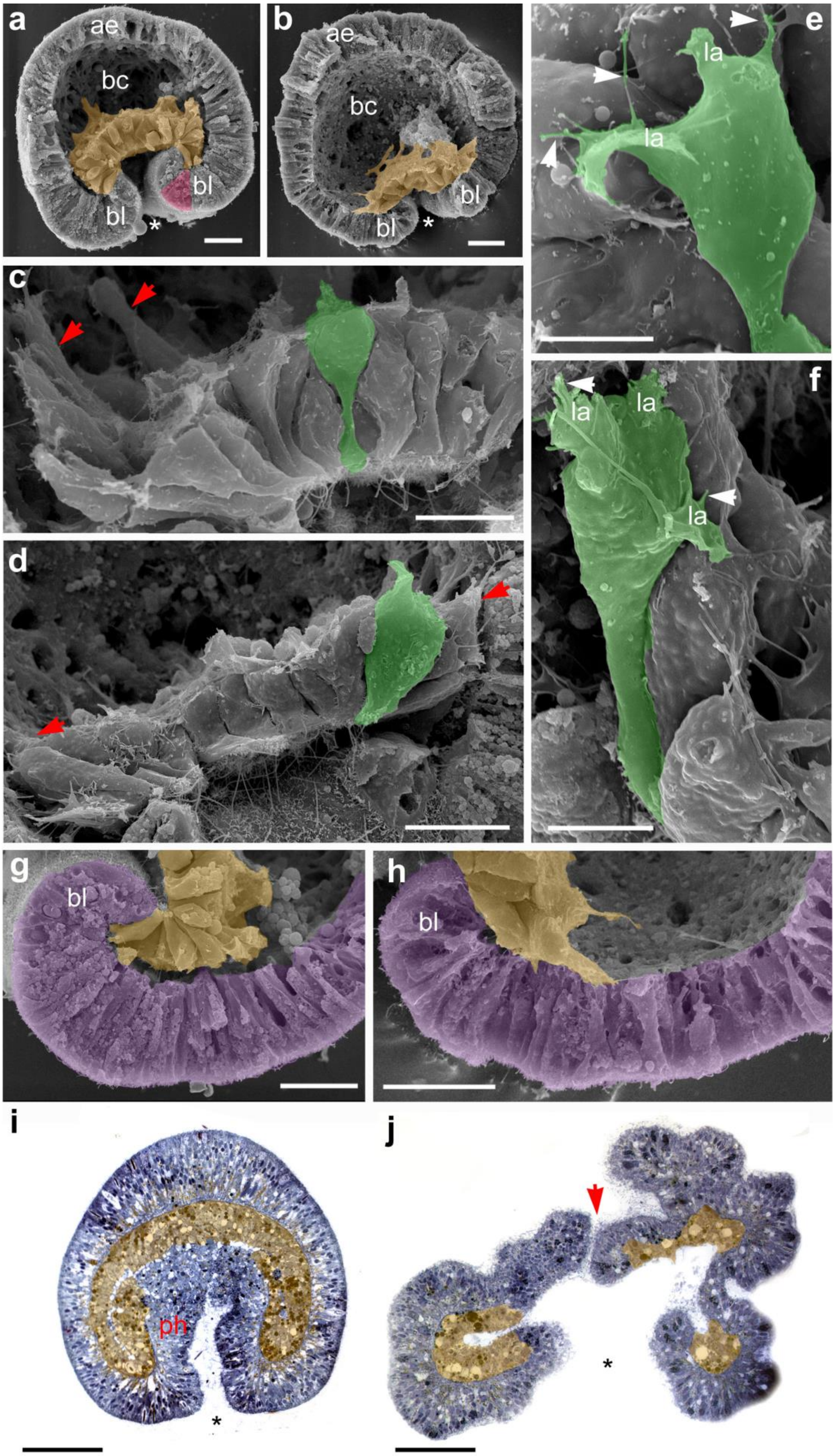
a
ae
bc
bl
bl
*
b
ae
bc
bl
bl
*
c
d
e
la
la
f
la
la
la
g
bl
h
bl
i
ph
*
j
*

Supplementary Figure 3.

Morphology of gastrulae and 72 hpf planulae subjected to DMSO or early AZK treatment.

24 hpf mid-gastrulae on **(a-h)** were split into halves along the oral-aboral axis prior to SEM; 72 hpf planulae on **(i, j)** were semithin sectioned along the oral-aboral axis prior to toluidine blue staining. **(a-b)** Overviews of the 24 hpf control **(a)** and 2.5 µM AZK treated **(b)** gastrulae. Pre-endodermal plates of both embryos (orange highlight) invaginate, although the pre-endodermal plate cells of the AZK treated embryo appear smaller. bl –blastopore lip; bc – blastocoel; ae – aboral ectoderm. **(c-d)** Pre-endodermal plate cells have typical bottle cell morphology both in DMSO **(c)** and in 2.5 µM AZK **(d)**. Red arrows point at the endodermal cells climbing up the basal surfaces of the ectodermal cells. In each plate one bottle cell is highlighted green. **(e-f)** The leading edges of the bottle cells in DMSO **(e)** and 2.5 µM AZK treated **(f)** gastrulae bear multiple protrusions typical for bottle cells of higher metazoans. la – lamellae; white arrows - filopodia. **(g-h)** SEM of the blastopore lip areas of the DMSO **(g)** and 2.5 µM AZK **(h)** treated embryo shows clear morphological difference between the columnar epithelial cells of the ectoderm wall (magenta highlight) and the bottle cells of the pre-endodermal plate (orange highlight). **(i-j)** DMSO treated 72 hpf planula **(i)** and a planula subjected to the early treatment with 5 µM AZK **(j)**; endoderm highlighted orange. Control planula **(i)** is teardrop-shaped and has a well-developed pharynx (ph); AZK treated planula **(j)** is flat, its blastopore re-opened to form a large hole, and an additional opening is visible in its aboral surface (arrow). Asterisks denote the blastopores. Scale bars: **a**, **b** - 30 µm; **c**, **d** - 20 µm; **e**, **f** - 10 µm; **g**, **h** - 30 µm; **i** - 50 µm; **j** - 20 µm.

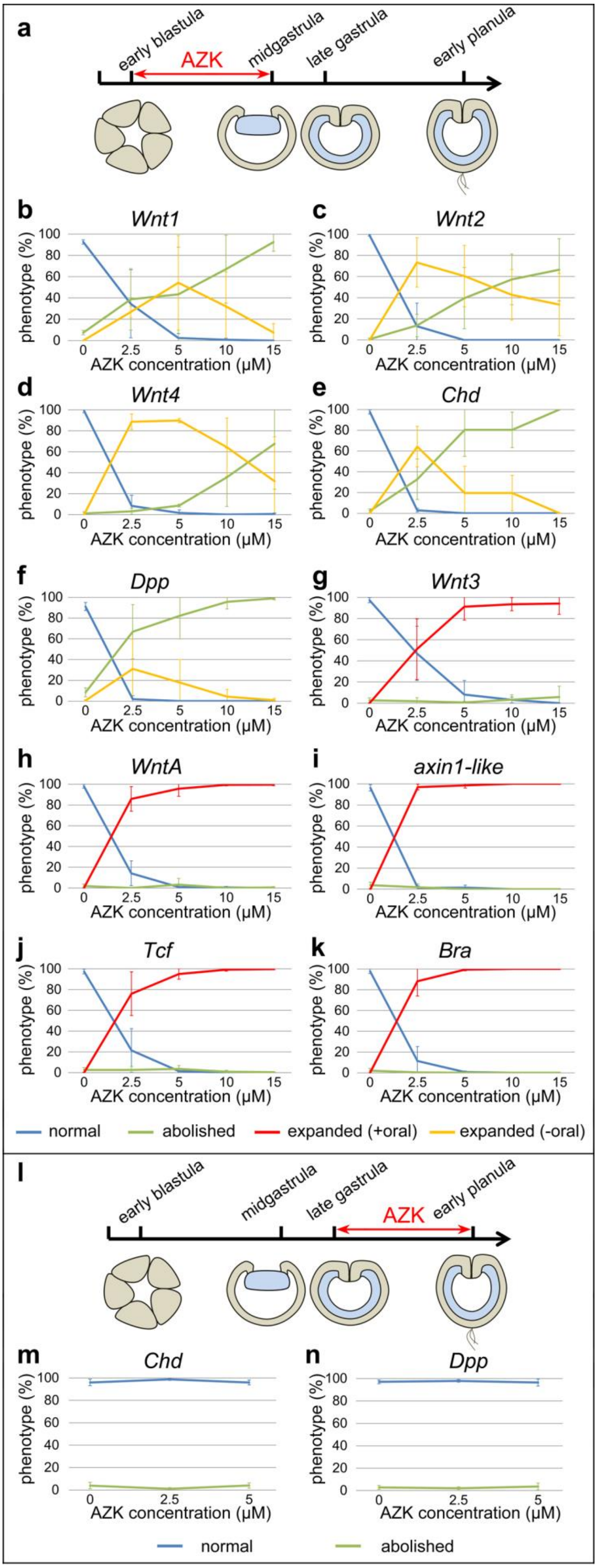
a
early blastula
AZK
midgastrula
late gastrula
early planula
b Wnt1
c Wnt2
d Wnt4
e Chd
f Dpp
g Wnt3
h WntA
i axin1-like
j Tcf
k Bra
phenotype (%)
AZK concentration (µM)
normal
abolished
expanded (+oral)
expanded (-oral)
l
early blastula
midgastrula
late gastrula
AZK
early planula
m Chd
n Dpp
phenotype (%)
AZK concentration (µM)
normal
abolished

Supplementary Figure 4.

Quantification of the penetrance of in situ hybridization phenotypes in AZK experiments.

Three independent experiments were performed for each gene and each AZK concentration. 0 µM AZK stands for DMSO control. The sample sizes for each experiment are presented in Supplementary Tables 1 and 2. Phenotypic categories: normal – wild type expression; abolished – no expression; expanded (-oral) – expression domain expands towards the aboral end and vacates the oral end of the embryo; expanded (+ oral) – expression domain expands towards the aboral end, but expression is retained at the oral end. Bars represent standard deviations . **(a-k)** Effect of early AZK treatment on the expression of the ectodermal *Wnt* genes, *Chd*, *Dpp*, *axin1-like*, *Tcf* and *Bra*. **(a)** Scheme of experiment. **(b-k)** Penetrance of the in situ hybridization phenotypes. **(l-n)** Effect of the late 2.5 µM AZK treatment on the expression of *Chd* and *Dpp*. **(l)** Scheme of experiment. **(m, n)** Penetrance of the in situ hybridization phenotypes.

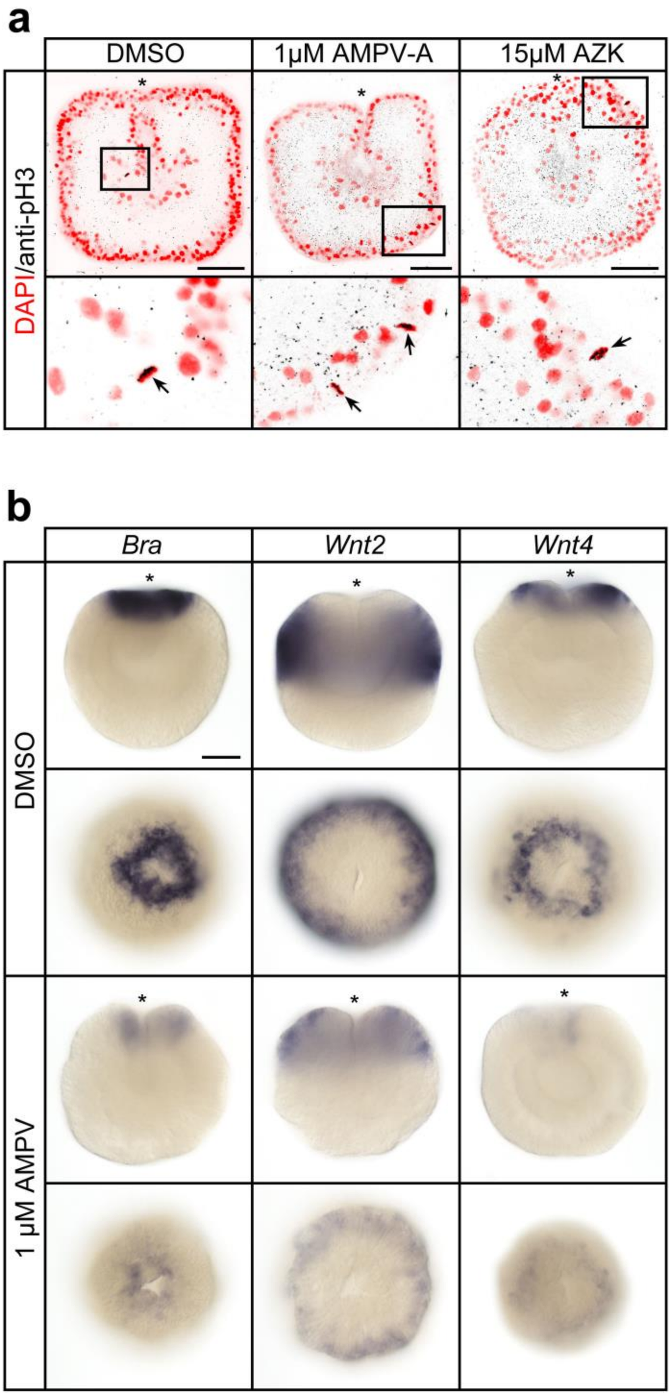

Supplementary Figure 5.

Expression domain changes observed in AZK are not due to inhibition of CDK1.

(a) DAPI and anti-phospho-Histone H3 antibody staining shows that metaphase plates are observed in the DMSO control, 15µM AZK and 1µM AMPV treated embryos. Areas boxed on top images are shown at higher magnification below. Arrows point at metaphase plates. Asterisks denote the blastopores. **(b)** Expression of *Wnt2*, *Wnt4* and *Bra* appears weaker in AMPV treated embryos than in control, but

expression domains remain the same. A lateral view (top, blastopore marked with an asterisk) and a corresponding oral view (bottom) is shown for each embryo. Scale bars: 50 µm.

Supplementary Tables

Supplementary Table 1. Sample size (number of embryos) in the early AZK treatment experiment.

Wnt1	DMSO	2,5µM AZK	5µM AZK	10µM AZK	15µM AZK
experiment 1	182	75	148	99	125
experiment 2	140	132	111	200	209
experiment 3	177	166	158	126	58

Wnt2	DMSO	2,5µM AZK	5µM AZK	10µM AZK	15µM AZK
experiment 1	134	224	191	197	202
experiment 2	159	206	234	154	176
experiment 3	227	110	110	122	245

Wnt3	DMSO	2,5µM AZK	5µM AZK	10µM AZK	15µM AZK
experiment 1	144	140	243	134	150
experiment 2	85	233	230	200	261
experiment 3	120	140	188	254	163

Wnt4	DMSO	2,5µM AZK	5µM AZK	10µM AZK	15µM AZK
experiment 1	187	214	177	205	116
experiment 2	134	324	210	216	249
experiment 3	105	111	195	197	162

WntA	DMSO	2,5µM AZK	5µM AZK	10µM AZK	15µM AZK
experiment 1	115	200	157	134	199
experiment 2	134	159	188	340	72
experiment 3	91	77	261	194	208

axin1-like	DMSO	2,5µM AZK	5µM AZK	10µM AZK	15µM AZK
experiment 1	85	288	99	188	221
experiment 2	152	170	257	236	228
experiment 3	208	198	171	77	118

Tcf	DMSO	2,5µM AZK	5µM AZK	10µM AZK	15µM AZK
experiment 1	166	120	83	159	199
experiment 2	113	172	285	225	355
experiment 3	120	290	131	152	189

Bra	DMSO	2,5µM AZK	5µM AZK	10µM AZK	15µM AZK
experiment 1	184	104	187	245	207
experiment 2	147	203	199	221	288
experiment 3	152	101	84	199	169

Chd	DMSO	2,5µM AZK	5µM AZK	10µM AZK	15µM AZK
experiment 1	137	108	188	134	153
experiment 2	197	189	236	195	229
experiment 3	157	80	125	164	184

Dpp	DMSO	2,5µM AZK	5µM AZK	10µM AZK	15µM AZK
experiment 1	197	217	140	227	156
experiment 2	175	209	221	220	216
experiment 3	179	68	173	153	142

Supplementary Table 2. Sample size (number of embryos) in the late AZK treatment experiment.

Chd	DMSO	2,5µM AZK	5µM AZK
experiment 1	112	190	141
experiment 2	136	140	161
experiment 3	182	249	139

Dpp	DMSO	2,5µM AZK	5µM AZK
experiment 1	99	226	115
experiment 2	116	146	104
experiment 3	134	205	228
